# Advancing kidney xenotransplantation with anesthesia and surgery - bridging preclinical and clinical frontiers challenges and prospects

**DOI:** 10.3389/fimmu.2024.1386382

**Published:** 2024-03-22

**Authors:** Xiaojian Zhang, Hailian Wang, Qin Xie, Yang Zhang, Yixin Yang, Man Yuan, Yuqi Cui, Si-Yuan Song, Jianzhen Lv, Yi Wang

**Affiliations:** ^1^ Central of Reproductive Medicine, Department of Obstetrics and Gynecology, Sichuan Academy of Medical Science and Sichuan Provincial People’s Hospital, University of Electronic Science and Technology of China, Chengdu, China; ^2^ Clinical Immunology Translational Medicine Key Laboratory of Sichuan Province, Sichuan Provincial People’s Hospital, University of Electronic Science and Technology of China, Chengdu, China; ^3^ Department of Critical Care Medicine, Zhongnan Hospital of Wuhan University, Wuhan, China; ^4^ Department of Anesthesiology, Perioperative and Pain Medicine, Stanford University School of Medicine, Standford, CA, United States; ^5^ The First Clinical Medical College of Norman Bethune University of Medical Sciences, Jilin, China; ^6^ Eye Center, Renmin Hospital of Wuhan University, Wuhan University, Wuhan, Hubei, China; ^7^ Department of Geriatrics, Donald W. Reynolds Institute on Aging, University of Arkansas for Medical Sciences, Little Rock, AR, United States; ^8^ Department of Neuroscience, Baylor College of Medicine, Houston, TX, United States; ^9^ School of Pharmacy, Guangxi University of Chinese Medicine, Nanning, China

**Keywords:** xenogeneic kidney transplantation, genetically modified pigs, anesthesia, immunological compatibility, organ rejection, immunotherapy, surgical techniques

## Abstract

Xenotransplantation is emerging as a vital solution to the critical shortage of organs available for transplantation, significantly propelled by advancements in genetic engineering and the development of sophisticated immunosuppressive treatments. Specifically, the transplantation of kidneys from genetically engineered pigs into human patients has made significant progress, offering a potential clinical solution to the shortage of human kidney supply. Recent trials involving the transplantation of these modified porcine kidneys into deceased human bodies have underscored the practicality of this approach, advancing the field towards potential clinical applications. However, numerous challenges remain, especially in the domains of identifying suitable donor-recipient matches and formulating effective immunosuppressive protocols crucial for transplant success. Critical to advancing xenotransplantation into clinical settings are the nuanced considerations of anesthesia and surgical practices required for these complex procedures. The precise genetic modification of porcine kidneys marks a significant leap in addressing the biological and immunological hurdles that have traditionally challenged xenotransplantation. Yet, the success of these transplants hinges on the process of meticulously matching these organs with human recipients, which demands thorough understanding of immunological compatibility, the risk of organ rejection, and the prevention of zoonotic disease transmission. In parallel, the development and optimization of immunosuppressive protocols are imperative to mitigate rejection risks while minimizing side effects, necessitating innovative approaches in both pharmacology and clinical practices. Furthermore, the post-operative care of recipients, encompassing vigilant monitoring for signs of organ rejection, infectious disease surveillance, and psychological support, is crucial for ensuring post-transplant life quality. This comprehensive care highlights the importance of a multidisciplinary approach involving transplant surgeons, anesthesiologists, immunologists, infectiologists and psychiatrists. The integration of anesthesia and surgical expertise is particularly vital, ensuring the best possible outcomes of those patients undergoing these novel transplants, through safe procedural practices. As xenotransplantation moving closer to clinical reality, establishing consensus guidelines on various aspects, including donor-recipient selection, immunosuppression, as well as surgical and anesthetic management of these transplants, is essential. Addressing these challenges through rigorous research and collective collaboration will be the key, not only to navigate the ethical, medical, and logistical complexities of introducing kidney xenotransplantation into mainstream clinical practice, but also itself marks a new era in organ transplantation.

## 1 Introduction

Kidney transplantation is thought of the gold standard treatment for those end-stage renal disease patients, which will present substantial improvement in quality of life and life expectancy. This procedure, particularly allogenic kidney transplantation, has achieved remarkable success in extending lives (1, 2). Nonetheless, the ongoing shortage of available organs for transplantation represents a significant hurdle, failing clinical demand. In response, the medical community has broadened the donor pool, such as utilizing organs from both deceased and living donors, including those marginal donors or undergoing ABO-incompatible transplants. Such efforts, while commendable, have not sufficiently mitigated the organ scarcity crisis. This situation underscores the urgent necessity for innovative strategies to augment the organ donor pool. Integrating advanced surgical techniques and anesthesia practices in the transplantation process is also vital, not only to ensure patient safety during these complex procedures but also to potentially increase the viability of organs from the marginal donors for transplantation. By refining surgical and anesthetic methodologies, the transplantation community can enhance post-operative outcomes and expand the criteria for donor acceptance, thereby addressing the pressing demand for kidney transplants (1).

Xenotransplantation, the process of transplanting organs or tissues between different species, has emerged as a pivotal solution in the quest to alleviate the organ shortage. Recent advancements in the realms of genetic engineering and immunosuppression, particularly through the use of targeted monoclonal antibodies, have propelled forward the research into kidney xenotransplantation. Experiments involving the transplantation of genetically modified porcine kidneys into non-human recipients have demonstrated significant progress, through those extended graft survival times reported (3). These achievements underscore the potential of xenotransplantation to bridge the gap in organ demand. However, translating these findings to human recipients is met with caution, due to the inherent biological and immunological differences, as well as the looming concerns over safety and ethical implications. The journey toward the clinical application of porcine-to-human kidney transplants is fraught with uncertainties, necessitating rigorous investigation and ethical deliberation.

The exploration of xenotransplantation, as a viable source of kidneys for transplantation, is gaining momentum, underpinned by the pressing need to address the organ shortage crisis. This approach not only promises to expand the donor pool but also to pioneer new frontiers in transplantation medicine (4). Advances in genetic modification techniques aim to enhance organ compatibility and reduce the likelihood of rejection and cross-species infection, marking a significant leap towards making xenotransplantation a reality for patients in need (4).

Moreover, the field of xenotransplantation is evolving alongside considerations of ethical standards and regulatory frameworks, ensuring that the development and potential clinical implementation are conducted with the utmost care for safety and ethical integrity. As the scientific community continues to unravel the complexities of xenogeneic organ transplantation, the collective goal remains clear: to provide a sustainable, ethical solution to the organ shortage crisis, ultimately saving more lives through innovative medical breakthroughs (5).

In essence, the path forward in xenotransplantation research and its prospective clinical application is laden with challenges yet brimming with potential. Through continued interdisciplinary collaboration, rigorous ethical scrutiny, and innovative scientific inquiry, xenotransplantation holds the promise of significantly impacting the future of organ transplantation, offering hope to countless individuals awaiting life-saving transplants.

## 2 Donor selection in kidney transplantation

In the advancement of xenogeneic kidney transplantation, the selection and genetic engineering of donor animals are paramount to ensuring the safety and efficacy of this innovative treatment approach. Due to the potential risk of virus transmission that makes non-human primates (NHPs) less suitable as xenotransplant donors, pigs have become the preferred source for donor organs. Pig kidneys, with their physiological and anatomical similarities to human kidneys, offer a practical alternative. They not only closely match human kidneys in size and function but also have the advantage of rapid reproduction and the capability for precise genetic modifications. However, the considerable genetic disparities between pigs and humans pose a significant challenge, often resulting in strong immune rejection and the failure of the transplant. To counteract these issues, genetic engineering plays a critical role.

**Figure 1:**
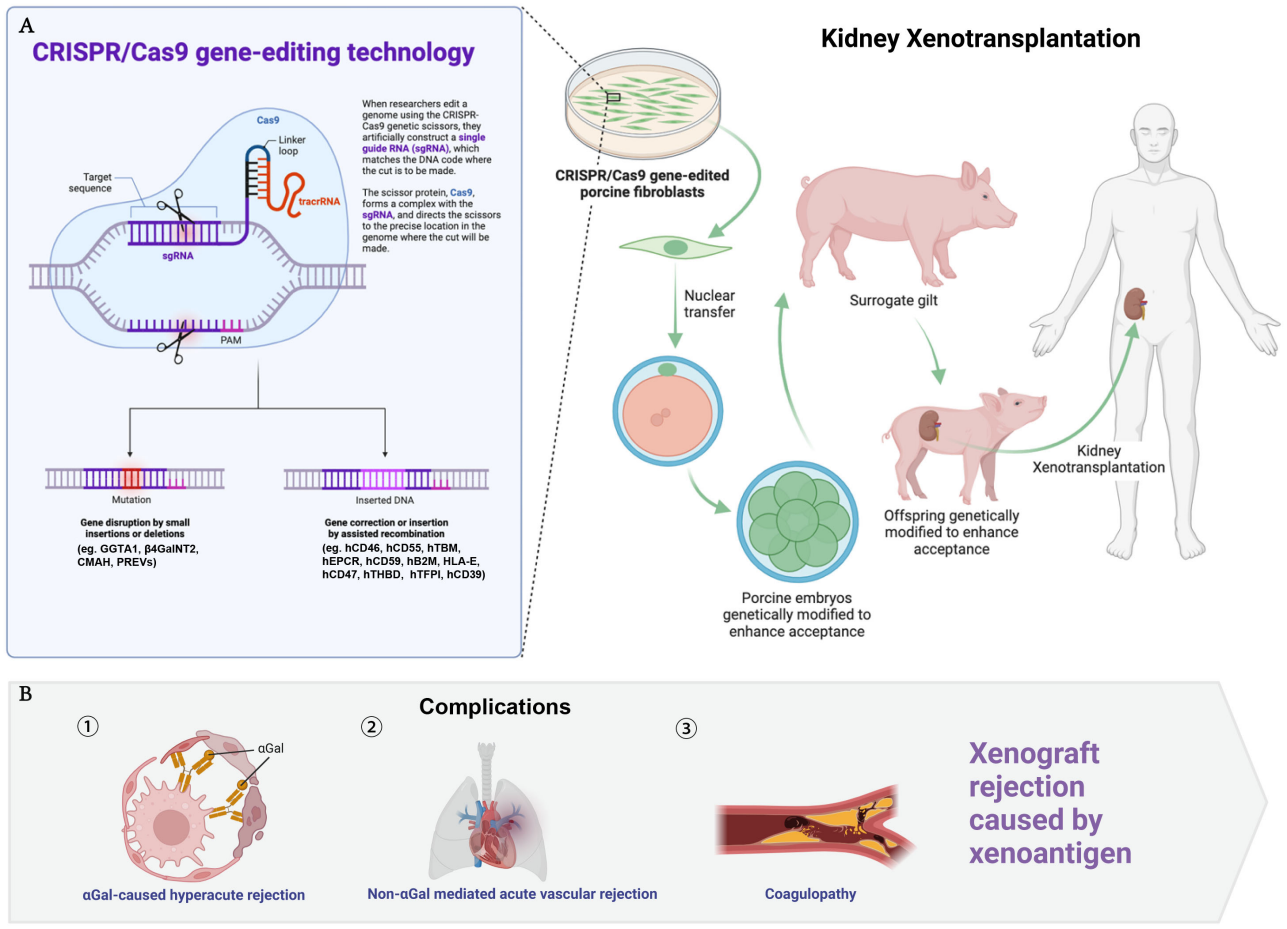
Genetic modifications in donor pigs for kidney xenotransplantation. This figure illustrates the CRISPR/Cas9 gene-editing technology and its application to donor pigs to enhance compatibility for kidney xenotransplantation **(A)**, while part B addresses the complications related to xenograft rejection **(B)**. The genetic modifications are listed as follows: GGTA1, alpha-1,3-galactosyltransferase; β4GalNT2, beta-1,4-N-acetylgalactosaminyltransferase 2; CMAH, cytidine monophosphate-N-acetylneuraminic acid hydroxylase; PREVs, porcine endogenous retroviruses, including PERV-A, PERV-B, PERV-C, inactivated to prevent cross-species viral transmission; hCD46, human membrane cofactor protein; hCD55, human decay-accelerating factor; hTBM, human thrombomodulin; hEPCR, human endothelial protein C receptor; hCD59, human protectin; hB2M, human beta-2 microglobulin; HLA-E, human leukocyte antigen E; hCD47, human integrin-associated protein; hTHBD, human thrombomodulin; hTFPI, human tissue factor pathway inhibitor; hCD39, human ectonucleoside triphosphate diphosphohydrolase 1.

The advent of CRISPR/Cas9 gene-editing technology has been a game-changer in this field. By targeting specific genes, scientists can reduce the risk of hyperacute and acute rejection responses. For instance, the elimination of the α-1,3-galactosyltransferase (GGTA1) gene in pigs prevents the expression of the αGal xenoantigen, significantly lowering the chances of hyperacute rejection. Further modifications, such as the deletion of the β-1,4-N-acetylgalactosaminyltransferase (β4GalNT2) and cytidine monophospho-N-acetylneuraminic acid hydroxylase (CMAH) genes, have been shown to mitigate acute vascular rejections that are not mediated by αGal antigens (6). The integration of human genes encoding complement regulatory proteins, anticoagulants, immune regulators, and other protective elements into the pig genome further enhances the compatibility of porcine kidneys with human recipients. These include genes for human complement regulatory proteins (hCD46 and hCD55), which help protect the transplanted organ from the recipient’s immune system, and genes like human thrombomodulin (hTBM) and human endothelial protein C receptor (hEPCR), which work to prevent clotting and improve graft survival (6–11) (**Figure 1**).

Research institutions, such as the University of Alabama, have made significant strides by utilizing pigs with a comprehensive suite of genetic modifications, including enhancements to immune regulation and coagulation profiles, as well as the removal of specific antigens known to trigger rejection (12). This meticulous genetic engineering aims to produce organs that are not only less likely to be rejected but also capable of performing their physiological functions without inducing harmful side effects in the recipient.

The continuous exploration and refinement of these genetic modifications underscore the complexity of making xenotransplantation a viable clinical option. As researchers dive deeper into the genetic underpinnings of immune rejection and organ compatibility, xenotransplantation alleviating the organ shortage crisis is coming true. Yet, it is essential to balance the enthusiasm for these technological advances with a careful assessment of their long-term implications for both organ recipients and the broader field of transplantation medicine.

Future research must also address the ethical considerations and regulatory challenges associated with xenotransplantation. As genetic engineering techniques become more sophisticated, consensus of the moral, ethical, and societal implications of using genetically modified animals for organ transplantation must be reached. Those topics warranting immediate attention includes animal welfare, the potential impact on natural ecosystems, and the long-term health outcomes for transplant recipients. Moreover, as the field progresses towards potential clinical applications, establishing clear and comprehensive regulatory frameworks will be crucial to ensuring the safety, efficacy, and ethical integrity of organ xenotransplantation.

While the genetic engineering of pigs for kidney xenotransplantation represents a frontier of medical science with the potential to dramatically expand the organ donor pool, it also poses a myriad of scientific, ethical, and regulatory challenges. Navigating these complexities will require concerted efforts from researchers, ethicists, and policymakers, with the ultimate goal of making xenotransplantation a safe, ethical, and effective solution for the millions worldwide in need of life-saving organ transplants.

## 3 Xenotransplantation recipient selection

Historically, xenotransplantation has traversed a challenging path, with initial attempts to transplant animal organs into humans dating back to the early 20th century, notably beginning in 1906 (13). The pursuit of understanding xenotransplantation’s complexities led researchers to utilize animal models that have smaller body sizes, despite their significant genetic differences from humans (e.g., a 33.4% nucleotide difference between mice and rats, compared to a 2.6% difference between macaques and baboons). These models, particularly rodents, were instrumental in elucidating the humoral and cellular dynamics of xenotransplantation. Up until 2012, rodents were the backbone of xenotransplantation studies, contributing to approximately 95% of research in the field. The advent of sophisticated genetic editing technologies has recently shifted the preference towards NHPs as models, given their closer genetic proximity to humans, which allows for more accurate simulation of immunosuppressive strategies and rejection mechanisms.

The utilization of brain-dead individuals as recipients of pig kidney transplants has served as a crucial intermediary step towards clinical xenotransplantation research (14). Although this model has provided invaluable insights, it is limited by the short survival times of the recipients, which restricts the exploration of long-term complications such as viral transmissions or abnormal organ growth (15). Establishing a standardized selection criterion for human recipients of xenotransplant organs remains a work in progress. Proposals suggest prioritizing individuals for whom conventional treatments have failed, or who face long waiting time for allogeneic transplants due to immunological sensitivities, such as a high level of panel reactive antibodies (PRA) (16). However, the selection of highly sensitized patients poses its own set of challenges, including the potential for adverse reactions between pig (swine leukocyte antigen, SLA) and human (human leukocyte antigen, HLA) antigens (16, 17). The field of xenotransplantation is at a critical juncture, requiring expanded research efforts to explore not only the scientific and ethical ramifications of these procedures but also the practical aspects of preparing for and managing post-transplant care. While the literature is currently sparse on the preoperative preparation and rehabilitation of recipients in the context of xenotransplantation, the lessons learned from allogeneic transplantation underscore the importance of comprehensive preoperative assessments and perioperative care. The clinical trials conducted at New York University and the University of Alabama at Birmingham involving xenotransplantation of kidneys into brain-dead human subjects have yielded valuable insights for perioperative management. Standard protocols for managing brain-dead patients before organ donation typically involve the administration of vasopressors, levothyroxine, steroids, and other interventions aimed at maintaining normal hemodynamics. During intraoperative anesthesia care, efforts mirror those in human-to-human kidney transplantation, encompassing administering immunosuppressive agents, maintaining metabolic stability, and optimizing hemodynamics to ensure adequate renal perfusion. To translate these findings into clinical practice, adherence to established standards of care is imperative. These pilot studies primarily addressed early-phase recovery, focusing on aspects such as hyperacute rejection, intraoperative life-threatening complications, and kidney function. Their findings lay the groundwork for future investigations to refine clinical practices in xenotransplantation (12, 18).. As xenotransplantation inches closer to clinical reality, the focus must also broaden to include patient rehabilitation and long-term care strategies that are tailored to the unique challenges of xenogeneic organ transplants. Additionally, the exploration of xenotransplantation as a bridge therapy for patients with end-stage renal disease presents an area ripe for investigation. This approach could potentially offer a lifeline to patients awaiting allogeneic transplants, with the added advantage of not precluding future allogeneic kidney transplants (19). The integration of xenotransplantation into the broader organ transplantation field raises complex questions regarding organ functionality, ethical considerations, and long-term patient outcomes. With kidneys playing a multifaceted role in human physiology, determining whether pig kidneys can fully meet human needs requires meticulous study. As research progresses, it will be crucial to develop clear guidelines for recipient selection, manage expectations regarding the outcomes of xenotransplantation, and ensure ethical standards are upheld in the pursuit of expanding the organ donor pool.

## 4 Challenges in kidney xenotransplantation

Xenotransplantation presents a multifaceted array of challenges, from immunological barriers to ethical considerations, each requiring meticulous attention to ensure the viability and success of organ transplants from pigs to humans.

The immunosuppressive regimen is pivotal in xenotransplantation, with a focus on minimizing the recipient’s immune response to the xenograft. Standard immunosuppressants such as tacrolimus, cyclosporine, mycophenolic acid (MPA), sirolimus, and corticosteroids form the cornerstone of current strategies. The evolution of immunosuppression has seen the introduction of novel agents targeting specific pathways critical for T-cell activation and the complement system. Anti-CD40 and anti-CD154 antibodies, for instance, have demonstrated potential in prolonging the survival of xenografts by inhibiting the CD40/CD154 co-stimulation pathway, a crucial step in T-cell mediated rejection (20, 21). Meanwhile, complement system inhibition, necessary to avert hyperacute rejection and thrombosis, relies on advanced strategies such as the use of C1 inhibitors and monoclonal antibodies like sutimlimab to suppress complement activation (22–24).

The physiological functions of pig kidneys, including erythropoietin (EPO) production and the regulation of the renin-angiotensin-aldosterone system (RAAS), along with maintaining proper acid-base balance, are critical for the graft’s integration and function within the human body (25, 26). The brain-dead decedents experience disrupted homeostasis and physiological functions, posing significant challenges even in critical care settings to preserve hemodynamic, hormonal, metabolic, and immune stability. In the initial pilot studies, standard intraoperative anesthesia and postoperative critical care protocols were implemented to ensure the maintenance of these vital parameters, mirroring established clinical practices (12, 18). The common complications include coagulopathy, stemming from endothelial damage and acute rejection, further complicate the post-transplant scenario, necessitating ongoing research and development of strategies to mitigate these effects.

Viral infections, particularly those associated with porcine endogenous retroviruses (PERV) and porcine cytomegalovirus (PCMV/PRV), pose significant risks for xenotransplantation. Nevertheless, both preclinical and clinical investigations conducted thorough pathogen screening to exclude prevalent viruses in pig donors, such as PERV-3 and PCMV. The post-transplantation virus detection in the decedents also remained negative (12, 27) However, the limitations of these negative findings are underscored by the relatively short observation periods. Consequently, developing future strategies to detect and eliminate these viruses is imperative to uphold the safety of both the graft and the recipient (28).

Additionally, the ethical landscape of xenotransplantation, encompassing animal rights, public attitudes, and regulatory milestones such as the FDA’s 2022 approval of a pig-to-human heart transplant, presents ongoing challenges (29). The pilot xenotransplantation trials have underscored various ethical and medicolegal considerations inherent in xenotransplantation research (12, 18). In these studies, brain-dead decedents were precluded from organ donation, and obtaining proper informed consent from their families was deemed essential. Notably, the absence of specific legislation governing xenotransplantation necessitates evolution of the regulatory frameworks to enhance research protocols and future clinical applications. Consultation with ethics committees is imperative to ensure adherence to established guidelines such as the Uniform Anatomical Gift Act (UAGA) and the dead-donor rule, while also acknowledging the cultural and religious nuances surrounding organ transplantation (30). Public acceptance and ethical considerations remain integral to the advancement and clinical application of xenotransplantation, highlighting the need for continued research, dialogue, and education in this evolving field.

In summary, xenotransplantation’s journey towards becoming a viable clinical option is fraught with complex immunological, physiological, virological, and ethical challenges. Each step forward requires a careful balance of innovation, safety, and ethical considerations, with the ultimate goal of expanding the organ donor pool and saving lives.

## 5 Conclusion and perspectives

Xenotransplantation has shown considerable promise in early-stage studies, bridging a critical gap between theoretical potential and practical clinical application (31). This transition from laboratory success to real-world efficacy underscores the importance of preclinical research as an essential step. This phase serves not only to validate findings from controlled laboratory settings in more clinically relevant scenarios but also to identify unforeseen challenges that may not be apparent in initial studies. The intricate dance between clinical application and laboratory research is informed by these challenges, directing the trajectory of future investigations. The exploration of genetically engineered pigs, particularly those modified with multiple genes, marks a significant advancement in this field (3). However, delving deeper into the specific functions of these genetic modifications and the discovery of new xenoantigens remain critical areas for further research. The protracted process of breeding these genetically altered pigs also poses a logistical challenge, emphasizing the need for streamlining breeding techniques to enhance research efficiency.

The ethical considerations surrounding the selection of participants for clinical trials, especially the inclusion of end-stage renal disease patients or those not eligible for conventional transplants, continue to provoke debate. Alternatively, the use of brain-dead individuals in preclinical studies presents a less contentious pathway, aligning with both ethical standards and research needs. Meanwhile, the quest for optimal immunosuppression strategies remains ongoing, with the current regimens requiring refinement to improve outcomes and reduce adverse effects. The consistency in selecting donor genotypes and standardizing perioperative care protocols also presents a significant hurdle, mirroring the complexity of translating xenotransplantation into a clinically viable option. Persistent issues such as graft rejection, inflammation, coagulation disorders, maintaining the physiological function of transplanted kidneys, and managing the risk of viral transmission underscore the multifaceted challenges ahead.

As preclinical research progresses, it is imperative to tackle these obstacles head-on, paving the way for the successful integration of xenotransplantation into clinical practice. The future of this innovative field hinges on our ability to navigate these complexities, requiring a concerted effort from researchers, clinicians, and ethicists alike. By addressing the nuanced challenges of genetic engineering, immunosuppression, and clinical trial design, xenotransplantation can move closer to becoming a tangible solution for the organ shortage crisis. Furthermore, enhancing the understanding of xenograft physiology and immunology will be crucial in developing targeted interventions that minimize rejection and improve long-term graft survival. Through these endeavors, xenotransplantation stands on the cusp of transitioning from an experimental procedure to a revolutionary treatment modality, offering hope to thousands of patients awaiting life-saving organ transplants.

## Author contributions

XZ: Writing – original draft. SS: Writing – original draft. HW: Writing – original draft. QX: Writing – original draft. YZ: Writing – original draft. YY: Writing – original draft. MY: Writing – original draft. YC: Writing – original draft. JL: Funding acquisition, Writing – review & editing. YW: Conceptualization, Funding acquisition, Investigation, Project administration, Validation, Writing – review & editing.
